# Design of peptide epitope from the neuraminidase protein of influenza A and influenza B towards short peptide vaccine development

**DOI:** 10.6026/97320630014183

**Published:** 2018-05-31

**Authors:** Sathish Sankar, Mageshbabu Ramamurthy, Subramanian Suganya, Balaji Nandagopal, Gopalan Sridharan

**Affiliations:** 1 Sri Sakthi Amma Institute of Biomedical Research, Sri Narayani Hospital and Research Centre, Sripuram, Vellore - 632055, Tamil Nadu, India

**Keywords:** Influenza virus, Neuraminidase, epitopes

## Abstract

Influenza viruses A and B are important human respiratory pathogens causing seasonal, endemic and pandemic infections in several parts of the globe with high morbidity and considerable mortality. The current inactivated and live attenuated vaccines are not effective. Therefore, it is of interest to design universal influenza virus vaccines with high efficacy. The peptide GQSVVSVKLAGNSSL of pandemic influenza, the peptide DKTSVTLAGNSSLCS of seasonal influenza and the peptide DILLKFSPTEITAPT of influenza B were identified as potential linear cell mediated epitopes. The epitopes predicted by BepiPred (B-cell epitope designer) program was subjected to docking experiment-using HexDock and CABS dock programs. The epitopes of pandemic H1N1 influenza A gave similar score of high affinity in docking. The epitope DKTSVTLAGNSSLCS of seasonal influenza A and epitope DILLKFSPTEITAPT of influenza B had high binding energy. It is further observed that the peptides GQSVVSVKLAGNSSL (pandemic influenza), DKTSVTLAGNSSLCS (seasonal influenza) DILLKFSPTEITAPT (influenza B) are found to interact with some known MHC class II alleles. These peptides have high-affinity binding with known MHC class II alleles. Thus, they have the potential to elicit cell immune response. These vaccines have to be further evaluated in animal models and human volunteers. These findings have application in the development of peptide B-cell epitope vaccines against influenza viruses.

## Background

An influenza virus poses a significant public health burden worldwide with morbidity of 3-5 million cases of severe illness. The estimate of financial encumbrance for the USA alone was over 100 billion dollars annually for influenza epidemic [1]. Worldwide, these annual epidemics due to seasonal influenza are estimated to result in about 3 to 5 million cases of severe illness, and about 290,000 to 650,000 deaths, as per WHO factsheet on seasonal influenza 2018 [2]. The 2009 H1N1 pandemic virus disproportionately affected children and young adults. Patients with chronic co-morbid illness, and those at the extremes of age and pregnant women are at higher risks of complications requiring hospitalization [3]. The 2009 H1N1 pandemic virus spread was so rapid that with 168 countries reported infections by mid-2009 [4] with more than 162,000 laboratory-confirmed cases and over a thousand human deaths [5]. Following this period, the 2009 H1N1 pandemic virus has subsequently caused seasonal epidemics along with influenza B viruses in most countries [6].

The current inactivated and live attenuated vaccines are not as effective as expected in the control of influenza as shown by recent reports [7]. This vaccination strategy is based on selection of specific vaccine strains annually. Due to antigenic drift, vaccines need to be reformulated every year to provide strain specific immunity, and this reformulation process is complex, expensive and time consuming especially for egg-adapted vaccines [8]. Several studies demonstrate efficacy of 75% with current seasonal influenza virus vaccines with decline in immunogenicity in the elderly [9]. Short protection duration, mismatches between vaccine strains and circulating strains being other factors associated with lower vaccine efficacy [10].

Towards the development of an improved vaccine design for seasonal influenza and for pandemic preparedness, several attempts are ongoing to design universal influenza virus vaccines [11]. One approach could be the development of multivalent peptide vaccine presenting linear peptide "exposed" B-cell epitopes from the consensus sequence of neuraminidase protein from influenza A and B viruses. The present study describes a significant advancement in this area. Such vaccines need to be evaluated in animal models and human volunteers.

## Methodology

### Sequence retrieval

All available complete amino acid sequence of neuraminidase gene from pandemic influenza H1N1 (n=758) and seasonal influenza (n=145) and influenza B (n=500) were retrieved from NCBI database as of December 2017.

### Consensus sequence

Consensus amino acid sequences each from pandemic and seasonal H1N1 influenza A and a consensus sequence for influenza B were identified using CLC Sequence Viewer 7 software program. The consensus sequence was used to identify Linear B cell epitopes from the predicted 3D model as shown below.

### Linear B-cell epitope prediction

The protein sequences were used to predict potential linear B-cell epitopes BepiPred 2 software program but NOT conformational epitope [12]. The epitope threshold was set at 0.5 as default parameter. The default score for epitope (E) is 0.5 in the program and changes in this alters sensitivity and specificity of the immunogenic efficacy of the epitope [13]. Positions above the threshold and of ~20-mer in length are considered as potential Bcell epitopes. Epitope positions, structural predictions (helix, sheet and coil) and surface accessibility (exposed and buried) of each epitope were taken into consideration by BepiPred program.

### 3D structure prediction using I-TASSER

I-TASSER online server program was used to predict 3D protein structure of neuraminidase protein of pandemic and seasonal influenza virus and influenza B virus. Consensus amino acid sequence was used for the prediction with default settings of the program. Among the 5 predicted models, the one that had high C-score was selected. C-score or confidence score estimates the quality of predicted models by I-TASSER that is calculated based on the significance of threading template alignments and the convergence parameters of the structure assembly simulations. Cscore typically ranges between -5 and 2, where a C-score of higher value signifies a model with a high confidence and viceversa [14].

### MHC-II binding predictions

MHC-II binding prediction was carried out for the epitopes as predicted by BepiPred. The online server program [15] was utilized using The Immune Epitope Database and Analysis Resource (IEDB) (recommended) prediction method and HLA allele reference set (a reference panel of 27 alleles) was used. The identified epitopes were sorted by percentile ranks. Good binders are indicated by lower percentile ranks. Three epitopes with least percentile ranks were chosen for molecular docking experiments.

This program has examined peptides and for each peptide, a percentile rank has been ascribed evaluating by three methods (combinatorial library, SMM_align and Sturniolo). The percentile rank is generated by comparing the peptide's score against the scores of five million random 15 mers selected from SWISSPROT database. A small numbered percentile rank indicates high affinity. The median percentile rank of the three methods was then used to generate the rank for consensus method. Peptides with median consensus percentile rank ≤ 20.0 are selected as predicted binders.

### Molecular Docking

#### Protein Preparation

The three dimensional structure of Human MHC II protein (PDB ID: 1AQD) was obtained from Protein Data Bank [16]. The receptor crystallographic water molecules were removed from the protein. Peptides were modeled in Pepfold using the PEPFOLD server [17]. The best 3D model was selected according to PEP-FOLD server, considering the lowest energy model that indicates peptide stability. The modeled peptides identified by BepiPred program were individually subjected to docking with the MHC class II protein Receptor, 1AQD using Hex 8.0.0. Protein docking program (http://hex.loria.fr), the Hex server is a first Fourier Transform (FFT) based analytics. In this method, rigid docking is undertaken taking into consideration different orientations through 6D analysis. The HEX program carries out a complete search over all six rigid-body degrees of freedom by rotating and translating the expansion coefficients [18, 19]. This was carried out by maintaining suitable parameters such as FFT mode-3D fast lite, grid dimension-0.6, receptor range-180, ligand range-180, twist range-360 and distance range-40. The docking experiments were further evaluated using CABS-dock server for protein-peptide docking. Docked complex of MHC haplotype DRB1 (PDB ID: 1AQD) with epitope were visualized in Pymol and corresponding interactions involved in binding were visualized in Discovery Studio Client 3.5. The CABS-dock online web server was used for docking with default server settings as described previously [20].

The high affinity MHC class II binding peptides (good binders) were used as ligands. The results were interpreted in terms of Cluster Density and Average Root Mean Square Deviation (RMSD) values. The density of the clusters (defined as an average difference between cluster elements divided by the number of elements) was used to rank the models. Ligand-RMSD (calculated for the peptides after superposition of receptor molecules) was used as the differentiation measure between cluster elements.

Ligand-RMSD (root mean square deviation calculated on the peptides after superposition of receptor molecules) was used as the differentiation measure between cluster elements. The following RMSD values were considered: High-quality prediction: RMSD < 3Å, Medium-quality prediction: 3 Å ≤ RMSD ≤ 5.5 Å, Low-quality prediction: RMSD > 5.5Å [21]. In the Hexdocking server 8.0 versions, more negative E-total value implied that there exists a strong interaction between ligand and receptor and that leads to activation of receptor activity.

## Results

### Linear B-epitope prediction

The predicted Linear B cell epitope for pandemic H1N1 influenza virus ranged from 11 to 37-mer in length. The two epitopes that were considered likely to be immunogenic were FAAGQSVVSVKLAGNSSLCPV (21-mer) with 70% exposed surface and 90% in the coiled region and GDNPRPNDKTGSCGPVSSN (19-mer) with 80% in the exposed surface and 100% in the coiled region. The MHC class-II cell receptor presents 11-mer or longer, the recognized "core" was nine residues long.

The predicted Linear B cell epitope for seasonal influenza H1N1 virus ranged from 11 to 20-mer in size. Two epitopes VAGEDKTSVTLAGNSSLCSI (20-mer) with 25% exposed surface and 95% in coiled region, GDNPRPEDGEGSCNPVTVD (19-mer) with 90% exposed surface and 100% in the highly coiled region and no epitope was recognized in the β-sheet or α-helix region.

The predicted linear B cell epitope for influenza B virus ranged from 11 to 32-mer in size, of which two epitopes of interest were SDILLKFSPTEITAPTMPL (19-mer) with 85% exposed surface and in the 100% coiled region followed by TKGVTLLLPEPEWTYPRLSCP (21-mer) with 62% exposed surface and in the 67% of coiled region and 33% in β sheet region.

### Protein 3D structure prediction using I-TASSER

**Figure 1:**
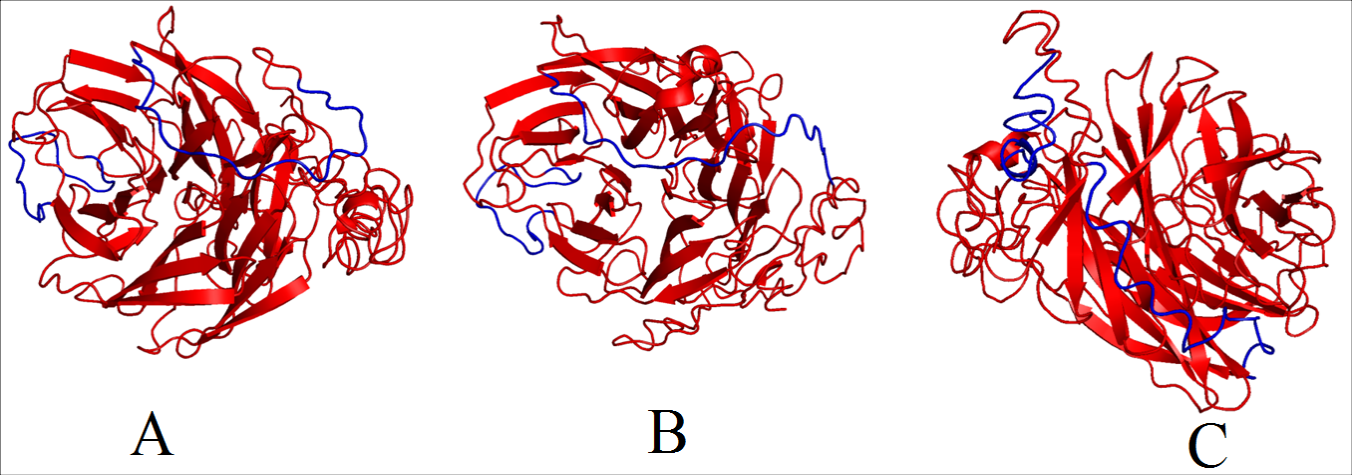
Predicted linear epitopes in the neuraminidase consensus sequence of H1N1 influenza pandemic (A), seasonal (B) and influenza B (C). The 3D ribbon structure of neuraminidase was generated using I-TASSER program and the epitopes are highlighted in blue color using the Pymol program for molecular visualization.

The model predicted by I-TASSER program for pandemic influenza had a C-score of 0.48 and RMSD score of 11.9. The model predicted for seasonal influenza had a C-score of 0.47 and RMSD score of 12.1. The model predicted for influenza B had a Cscore of -1.40 and RMSD score of 10.4. The predicted linear B cell epitopes were highlighted in the neuraminidase protein structure of pandemic and seasonal H1N1 influenza A (Figure 1A and B) and Influenza B (Figure 1C). These models were chosen for the identification and location of the epitopes.

### MHC Class-II binding predictions

Results of MHC Class-II binding predictions of the linear B-cell epitopes on neuraminidase as predicted by BepiPred program are shown in Table 1. Six epitopes with least percentile ranks are listed each for influenza A H1N1 (pandemic and seasonal) and influenza B. Between the two epitopes of pandemic H1N1 virus, FAAGQSVVSVKLAGNSSLCPV had a lesser percentile rank compared to the GDNPRPNDKTGSCGPVSSN. Similarly, between the two epitopes of seasonal H1N1 virus, VAGEDKTSVTLAGNSSLCSI had a lesser percentile rank compared to GDNPRPEDGEGSCNPVTVD. Similarly, for Influenza B, between the two epitopes, SDILLKFSPTEITAPTMPL had the lesser percentile rank compared to TKGVTLLLPEPEWTYPRLSCP.

### Molecular Docking

Two epitopes each from influenza A (pandemic and influenza) and influenza B were selected that had lower percentile ranks in BepiPred program and subjected to docking experiment using HexDock and CABS dock program. Both the epitopes of pandemic H1N1 influenza A gave similar score of high affinity in HEX dock program. The epitope DKTSVTLAGNSSLCS of seasonal influenza A and epitope DILLKFSPTEITAPT of influenza B had high binding energy in terms of E-score (Table 2). The interactions of amino acid residues for the three docked protein-ligand complex. The peptide GQSVVSVKLAGNSSL of pandemic influenza with the MHC class II protein interacting residues were Asp A: 27, Val A: 6,Val A: 91,Thr A: 93. The peptide DKTSVTLAGNSSLCS of seasonal influenza with the MHC class II protein interacting residues was Glu A: 141. The peptide DILLKFSPTEITAPT of influenza B with the MHC class II protein interacting residues were Thr A: 93, Val A: 91, Thr A: 90,Asp A: 110, Arg A: 140, Glu A: 141.

## Discussion

Influenza viruses continue to cause substantial morbidity and mortality worldwide due to the relative role of epidemic dynamics, viral evolution, and climatic drivers [22, 23]. Current influenza vaccines mostly aim at the induction of specific neutralizing antibodies. These vaccines depend upon the predicted circulating strains. As a consequence of frequent mismatches that occur among circulating strains, only vaccine with suboptimal efficacy is generated. The development of "universal" influenza virus vaccines that induces broadly neutralizing antibodies is still being explored [24].

This study was focused on the development of neuraminidasebased B-cell peptide epitope vaccine to elicit good antibody response against divergent antigenic types (pandemic and seasonal influenza A and influenza B). We have developed a consensus amino acid sequence moiety for identifying B-cell epitopes and carried out affinity determination and molecular docking. Antibodies to the neuraminidase protein primarily aggregate virus on the cell surface, and thus reduces the amount of virus release from infected cells efficiently. Many antiviral drugs that target sialic acid binding that blocks neuraminidase enzyme activity are in use [25]. Compared to haemagglutinin, a dominant protective response is targeted towards more antigenic neuraminidase and reported as a good inducer of crossprotecting immunity [26]. Therefore we anticipated that the peptide epitope vaccine directed against conserved regions of neuraminidase protein could elicit protective and strong immune response. The secretory IgA antibodies in the respiratory tract would block the spread of the virus efficiently and attenuate the disease or abort the infection [27, 28].

Influenza live attenuated vaccine was used during 2015-2016 but the effectiveness was not acceptable in children [29]. The recommended trivalent vaccines for use in the 2016-2017 influenza season (northern hemisphere winter) contained an A/California/7/2009 (H1N1) pdm09-like virus; an A/Hong Kong/4801/2014 (H3N2)-like virus a B/Brisbane/60/2008-like virus. Also, a quadrivalent vaccine was recommended which contained the above three viruses and the B/Phuket/3073/2013- like virus. The same was recommended for southern hemisphere according to WHO factsheet report, 2015. Previously, 13 novel substitutions have been identified which affected vaccine efficacy due to antigenic drift [30].

We in our study used a consensus sequence of neuraminidase protein amino acid sequence to identify candidate linear B-cell peptide epitopes for vaccine development. Previously, a peptidebased vaccine directed against conserved parts of influenza virus containing B and T cell epitopes was reported [31]. We did not predict conformational epitope due to technical complications in design and synthesis where the conformation due to mutations changes every year. As we focussed on linear B-cell epitopes, only MHC Class II binding was evaluated. This study does not focus on T-cell epitopes.

Xu et al. [32] identified highly conserved and subtype-specific peptide epitopes within each of N1, N2 and type B neuraminidase proteins using Geneious 7.0.6 software program. These peptides were shown to generate mono-specific antibodies against their respective subtype/type in experimental rabbits. The synthetic peptides linked to a 6-aminocaproic-cysteine and conjugated to a Keyhole limpet hemocyanin (KLH) carrier protein. The KLH was shown to be an ideal carrier protein for peptide vaccines applied to humans. Previously, a similar approach has been taken for developing human cancer vaccines [33].

Immune response to peptides is influenced by the way they are presented to the immune system, and therefore a multifunctional delivery systems coupling the antigen with adjuvant is needed [34]. This approach has been addressed subsequently by many researchers and has shown similar effects [32]. Gold nanoparticles mediated OVA peptide delivery [35], peptides adsorbed on poly (D, L-lactide-co-glycolide) (PLGA) particles as a controlled-release vaccine delivery system [36]. A novel liposome has been developed to deliver peptides, surface modified by 3-methyl-glutarylated hyperbranched poly (glycidol) (MGlu-HPG), to enhance antigen-specific immunity in vitro and in vivo and to function as a vaccine carrier [37].

The MHC class-II cell receptor presents 15-mer or longer [38]. Peptides binding to class II proteins are not constrained in size and can vary from 11 to 30 amino acids long. The peptidebinding groove in the MHC class II molecules is open at both ends, which enables binding of peptides with relatively longer length. Though the "core" nine residues long segment contributes the most to the recognition of the peptide, the flanking regions are also important for the specificity of the peptide to the class II allele [39]. The BepiPred program identified immunogenic B-cell epitopes of neuraminidase genes of influenza A and B. From the pandemic Influenza a consensus neuraminidase sequence, FAAGQSVVSVKLAGNSSLCPV (21-mer) and GDNPRPNDKTGSCGPVSSN (19-mer) were identified. In the case of seasonal influenza, the predicted Linear B cell epitope for H1N1 virus ranged from 11 to 20-mer in size. Two epitopes VAGEDKTSVTLAGNSSLCSI (20-mer) with 25% exposed surface and GDNPRPEDGEGSCNPVTVD (19-mer) with 90% exposed surface.

Likewise, for influenza B virus, the predicted Linear B cell epitopes were SDILLKFSPTEITAPTMPL (19-mer) with 85% exposed surface and TKGVTLLLPEPEWTYPRLSCP (21-mer) with 62% exposed surface. Present 11-mer knows the MHC class- II cell receptor or longer, the recognized "core" is nine residues long [39]. In general B-cell epitope are flat, oblong, oval shaped volume containing mainly hydrophobic amino acids in the center flanked by charged residues. The average epitope is made up of about 15 residues with one linear stretch of 5 or more residues constituting more than half of the epitope size [40].

Tewawong et al. [41] reported B-cell epitopes using BepiPred 1 program in influenza A and B viruses at different amino acid positions. So, we chose to build a consensus sequence from all available GenBank sequences of influenza strains and use these sequences for BepiPred 2 program. The identified B-cell epitopes of influenza A virus were in the most conserved region. Jagadesh et al reported the conserved regions [42]. The epitope identified for influenza B DILLKFSPTEITAPT was subjected to BLAST analysis and found to have 100% homology only to influenza B virus strains (n=100) reported from several countries as of 27 February 2018.

Using the consensus amino acid sequences of neuraminidase protein of pandemic and seasonal influenza A and B, 3D protein model was obtained from I-TASSER program. The position of the epitopes was determined using Pymol program. The same sequence was also submitted for I-TASSER to identify the location of the epitopes in the native neuraminidase molecule. The exposed or buried nature of the epitopes was identified in the model. We found one exposed epitope each on neuraminidase protein of pandemic and seasonal influenza A and influenza B. It could be postulated that the antibody elicited to such exposed epitopes would not be subjected to steric hindrance and hence antibody would interfere neuraminidase function [43]. Thus the antibody elicited by the peptide would reduce the influenza virus infectivity in the respiratory tract.

Computational docking was carried out to confirm the binding of MHC class II protein with selected B cell epitopes. Docking programs represent structure of receptor-ligand complex in terms of lowest free energy state [44].

A critical step in CD4+ T cell activation is the recognition of B cell epitopes presented by MHC class II molecules [45]. The class II binding groove is open at both ends and therefore, peptides binding to class II molecules tend to be typically between 13 and 25 amino acid residues in length [46]. Several B-cell epitopes have been previously investigated using various proteins-protein docking methods such as PatchDock [47], HEXdock [48] and CABSDock [18]. We used PEPFOLD server to model the peptides. In docking experiments we used both HEX dock and CABS Dock. Binding affinity in terms of free energy and interacting residues were identified from HEXdock program and the RMSD score were identified from CABSdock program. To be able to analyze the docking, the e-values have obtained using the Hex software. The docking process is considered more efficient, when the e-values are low. Hex's docking calculations encodes both surface shape and electrostatic charge and potential distributions for each molecule as modeled by the 3D expansions of real orthogonal spherical co-ordinates (its coordinate surfaces x = constant, y = constant, and z = constant are planes that meet at right angles to one another).

In our study, among the HLA allele reference set (HLA DR, DQ, DP) used to screen the potential linear B-cell epitopes, HLADRB1 was found to have the highest binding affinity with the epitopes identified by BepiPred program for both influenza A and B. Hence, HLA-DRB1 was chosen as receptor protein and the two epitopes identified to have high affinity for docking.

In this study, immunoinformatics approaches were employed to design an efficient multi-epitope peptide vaccine consisting of immunogenic Linear B epitopes from both influenza A (pandemic and seasonal) and influenza B viruses. As alluded to earlier in the discussion, it is now possible to deliver such multipeptide vaccines to elicit a good immune response.

## Conclusion

There is a need to develop efficient vaccines against emergent antigenic types of influenza A and B viruses. We report three specific peptides such as the GQSVVSVKLAGNSSL (pandemic influenza), DKTSVTLAGNSSLCS (seasonal influenza) and DILLKFSPTEITAPT (influenza B) as potential linear epitopes for a candidate peptide vaccine.

## Figures and Tables

**Table 1:** Results of MHC Class-II binding predictions of the linear B-cell epitope as predicted by BepiPred program.

HLA Allele (reference set)	Peptide	Method Used	Percentile rank
Peptide: FAAGQSVVSVKLAGNSSLCPV (pandemic)			
DRB1*08:02	AGQSVVSVKLAGNSS	Consensus (smm/nn/sturniolo)	2.09
DRB1*08:02	GQSVVSVKLAGNSSL	Consensus (smm/nn/sturniolo)	2.09
DRB1*08:02	QSVVSVKLAGNSSLC	Consensus (smm/nn/sturniolo)	2.09
DRB1*08:02	SVVSVKLAGNSSLCP	Consensus (smm/nn/sturniolo)	2.09
DRB1*08:02	VVSVKLAGNSSLCPV	Consensus (smm/nn/sturniolo)	2.12
DRB1*08:02	AAGQSVVSVKLAGNS	Consensus (smm/nn/sturniolo)	2.31
GDNPRPNDKTGSCGPVSSN (pandemic)			
DQA1*05:01/DQB1*03:01	RPNDKTGSCGPVSSN	Consensus (comb.lib./smm/nn)	21.87
DQA1*05:01/DQB1*03:01	PRPNDKTGSCGPVSS	Consensus (comb.lib./smm/nn)	23.8
DQA1*05:01/DQB1*03:01	NPRPNDKTGSCGPVS	Consensus (comb.lib./smm/nn)	49.93
DRB1*09:01	NPRPNDKTGSCGPVS	Consensus (comb.lib./smm/nn)	55.3
DRB1*09:01	PRPNDKTGSCGPVSS	Consensus (comb.lib./smm/nn)	55.3
DRB1*09:01	RPNDKTGSCGPVSSN	Consensus (comb.lib./smm/nn)	55.3
VAGEDKTSVTLAGNSSLCSI (Seasonal)			
DRB1*11:01	KTSVTLAGNSSLCSI	Consensus (smm/nn/sturniolo)	3.79
DRB1*11:01	DKTSVTLAGNSSLCS	Consensus (smm/nn/sturniolo)	3.94
DRB1*04:01	KTSVTLAGNSSLCSI	Consensus (smm/nn/sturniolo)	4.34
DRB1*04:01	DKTSVTLAGNSSLCS	Consensus (smm/nn/sturniolo)	4.47
DRB1*04:01	GEDKTSVTLAGNSSL	Consensus (smm/nn/sturniolo)	8.45
DRB1*04:01	EDKTSVTLAGNSSLC	Consensus (smm/nn/sturniolo)	8.83
GDNPRPEDGEGSCNPVTVD (Seasonal)			
DQA1*05:01/DQB1*03:01	PRPEDGEGSCNPVTV	Consensus (comb.lib./smm/nn)	31.48
DQA1*05:01/DQB1*03:01	RPEDGEGSCNPVTVD	Consensus (comb.lib./smm/nn)	31.85
DQA1*05:01/DQB1*03:01	NPRPEDGEGSCNPVT	Consensus (comb.lib./smm/nn)	36.71
DQA1*05:01/DQB1*03:01	GDNPRPEDGEGSCNP	Consensus (comb.lib./smm/nn)	46.46
DQA1*05:01/DQB1*03:01	DNPRPEDGEGSCNPV	Consensus (comb.lib./smm/nn)	46.46
DQA1*03:01/DQB1*03:02	RPEDGEGSCNPVTVD	Consensus (comb.lib./smm/nn)	46.61
SDILLKFSPTEITAPTMPL (Influenza B)			
DRB1*09:01	SDILLKFSPTEITAP	Consensus (comb.lib./smm/nn)	0.68
DRB1*09:01	DILLKFSPTEITAPT	Consensus (comb.lib./smm/nn)	0.93
DRB1*09:01	ILLKFSPTEITAPTM	Consensus (comb.lib./smm/nn)	1.69
DRB1*15:01	SDILLKFSPTEITAP	Consensus (smm/nn/sturniolo)	2.01
DRB1*15:01	DILLKFSPTEITAPT	Consensus (smm/nn/sturniolo)	2.96
DRB1*07:01	SDILLKFSPTEITAP	Consensus (comb.lib./smm/nn)	3.08
TKGVTLLLPEPEWTYPRLSCP (Influenza B)			
DRB1*04:05	TKGVTLLLPEPEWTY	Consensus (smm/nn/sturniolo)	2.14
DRB1*04:05	KGVTLLLPEPEWTYP	Consensus (smm/nn/sturniolo)	2.54
DQA1*04:01/DQB1*04:02	TKGVTLLLPEPEWTY	Consensus (comb.lib./smm/nn)	2.93
DQA1*04:01/DQB1*04:02	KGVTLLLPEPEWTYP	Consensus (comb.lib./smm/nn)	4.27
DRB1*04:05	GVTLLLPEPEWTYPR	Consensus (smm/nn/sturniolo)	4.7
DRB1*04:05	VTLLLPEPEWTYPRL	Consensus (smm/nn/sturniolo)	7.06

**Table 2:** Results of molecular docking (PDB ID: 1AQD), DRB1 (MHC class II haplotype) with B-cell epitopes showing good binding.

Epitope	CABS DOCK			Docking score HEX 8.0 version
Cluster density	Ave RMSD	Max RMSD	E-Score
AGQSVVSVKLAGNSS	114.558	0.881	10.4599	-470.84
GQSVVSVKLAGNSSL	39.2913	1.80702	22.8967	-470.84
KTSVTLAGNSSLCSI	72.113	1.38671	2.74745	-464.24
DKTSVTLAGNSSLCS	37.122	3.04378	21.374	-504.26
SDILLKFSPTEITAP	40.0217	5.14721	35.8884	-509.75
DILLKFSPTEITAPT	35.676	3.72797	11.7607	-521.78
CABS: Carbon Alpha (Cα), carbon Beta and the Side-chain Dock. The three pseudo-atoms represent each amino acid residue. E value: Energy of docking. The HEX program carries out a complete search over all six rigid-body degrees of freedom by rotating and translating the expansion coefficients. RMSD: Root Mean Square Deviation.
